# Bluues: a program for the analysis of the electrostatic properties of proteins based on generalized Born radii

**DOI:** 10.1186/1471-2105-13-S4-S18

**Published:** 2012-03-28

**Authors:** Federico Fogolari, Alessandra Corazza, Vijaylakshmi Yarra, Anusha Jalaru, Paolo Viglino, Gennaro Esposito

**Affiliations:** 1 Dipartimento di Scienze Mediche e Biologiche. Università di Udine, Piazzale Kolbe, 4, Udine 33100, Italy; 2 Istituto Nazionale Biostrutture e Biosistemi, Viale medaglie d'Oro 305, Roma 00136, Italy

## Abstract

**Background:**

The Poisson-Boltzmann (PB) equation and its linear approximation have been widely used to describe biomolecular electrostatics. Generalized Born (GB) models offer a convenient computational approximation for the more fundamental approach based on the Poisson-Boltzmann equation, and allows estimation of pairwise contributions to electrostatic effects in the molecular context.

**Results:**

We have implemented in a single program most common analyses of the electrostatic properties of proteins. The program first computes generalized Born radii, via a surface integral and then it uses generalized Born radii (using a finite radius test particle) to perform electrostic analyses. In particular the ouput of the program entails, depending on user's requirement:

1) the generalized Born radius of each atom;

2) the electrostatic solvation free energy;

3) the electrostatic forces on each atom (currently in a dvelopmental stage);

4) the pH-dependent properties (total charge and pH-dependent free energy of folding in the pH range -2 to 18;

5) the pKa of all ionizable groups;

6) the electrostatic potential at the surface of the molecule;

7) the electrostatic potential in a volume surrounding the molecule;

**Conclusions:**

Although at the expense of limited flexibility the program provides most common analyses with requirement of a single input file in PQR format. The results obtained are comparable to those obtained using state-of-the-art Poisson-Boltzmann solvers. A Linux executable with example input and output files is provided as supplementary material.

## Background

### Generalized Born models

Electrostatic effects arising due to the interaction of solute charges among themselevs and with solvent and ion charges, are of utmost importance for biomolecular structure and function. Continuum methods based on the Poisson-Boltzmann equation have been widely used for calculating electrostatic effects [1-5]. In the last decades much interest has been devoted to developing fast approximations to the solution of the Poisson-Boltzmann (PB) equation.

Onufriev and coworkers have developed an analytical approximation to the exact potential inside and outside the low dielectric region of a sphere [6], that performs surprisingly well also for the complex shape of proteins [7-10] and is therefore more general than the generalized Born (GB) models.

When only self- and interaction energies and forces are sought, the most widely used approach is based on generalized Born (GB) models. Recent reviews summarize the approach and highlight most interesting recent results [3,11-14].

Central to these models is the estimation of polarization charge contributions to: i) the self-energy of each charge (embedded in the solute); ii) the interaction energy of each pair of charges.

By equating the estimated reaction field ${Û}_{i}^{react}$ at the *i_th _*charge *q_i _*to the reaction field at a spherical ion in solution with the same charge, each charge is assigned an effective radius which is called the Born radius (*α_i_*).

(1) $$\alpha_{i}=\frac{1}{{Û}_{i}^{react}}\frac{q_{i}}{4\pi \varepsilon_{0}}\left(\frac{1}{\varepsilon_{out}}-\frac{1}{\varepsilon_{in}}\right)$$

Once Born radii have been estimated the interaction energy between any two charges is computed as the sum of a direct Coulomb term (computed using the solute dielectric constant) and a solvation term which is, according to Still et al. [15], $\Delta G_{solv}=\sum_{i,j}\Delta G_{solv,ij}$ with:

(2) $$\Delta G_{solv,ij}=-\frac{1}{8\pi }\left(\frac{1}{\varepsilon_{in}}-\frac{1}{\varepsilon_{out}}\right)\frac{q_{i}q_{j}}{\sqrt{r_{ij}^{2}+\alpha_{i}\alpha_{j}\exp^{\frac{-r_{ij}^{2}}{4\alpha_{i}\alpha_{j}}}}}$$

This expression was found to be more accurate than the exact expression for two charges embedded in a conducting sphere, although the coefficient of 8.0 instead of 4.0 in the exponential has been suggested [16] and other similar forms have been proposed [17,18].

### Computation of Born radii

Born radii could be in principle computed by solving a system of linear equations for the polarization charges at the boundaries of the solute volume [19-25]. Under the approximation that the ionic solution provides complete screening, amounting to the assumption that the surface behaves like a grounded conductor, polarization charges at the surface are such that the integral of their electric field at any outer surface point is exactly the opposite of the source charge field. This approximation amounts to setting *ε_out _*= ∞. In a different context the same approximation has been proposed many years ago as the conducting surface model (COSMO) [26,27] and successfully used since then. Under this condition it is possible to obtain simple formulae for the generalized Born radius for a low dielectric region delimited by a sphere or a plane. The reaction field satisfies Laplace equation inside the surface and the solution may be obtained solving the equation using suitable basis functions with Dirichlet boundary conditions at discretized points on the surface [28].

These methods are however slower than approximate methods. The computation of Born radii is performed by volume or surface integrals. An approximation that has been widely used is the Coulomb field approximation, where the formula for the electrostatic displacement for a uniform medium is applied in the inner and outer regions using the inner and outer dielectric constant, respectively. Born radius in this approximation does not depend on the inner and outer dielectric constant, but rather becomes a purely geometric quantity. The volume integral formulation of the Coulomb field approximation is turned into a surface integral formulation using the divergence theorem [29]. The Coulomb field approximation corresponds to neglecting the contribution to polarization at the surface due to polarization charges and to considering therefore the electric field inside the surface as due only to the source charge:

(3) $$\vec{E}\left({\vec{r}}_{S}\right)=\frac{q_{i}}{4\pi \varepsilon_{0}\varepsilon_{in}}\times \frac{\left({\vec{r}}_{S}-{\vec{r}}_{i}\right)}{||{\vec{r}}_{S}-{\vec{r}}_{i}|{|}^{3}}$$

Under the assumption of grounded conducting surface the outer field is zero and the polarization charge at the surface point ${\vec{r}}_{S}$ is given by Gauss' theorem:

(4) $$\sigma \left({\vec{r}}_{S}\right)=-\frac{q_{i}}{4\pi }\times \frac{\left({\vec{r}}_{S}-{\vec{r}}_{i}\right)\cdot \vec{n}\left({\vec{r}}_{S}\right)}{||{\vec{r}}_{i}-{\vec{r}}_{S}|{|}^{3}}$$

where $\vec{n}\left({\vec{r}}_{S}\right)$ is the unit vector normal to the surface at point ${\vec{r}}_{S}$ and pointing outwards.

The reaction field at the source charge will be equal to the integral of the fields due to surface charges at the source charge, i. e.:

(5) $$U_{i}^{react}=\int_{S}\frac{\sigma \left({\vec{r}}_{S}\right)}{4\pi \varepsilon_{0}\varepsilon_{in}||{\vec{r}}_{i}-{\vec{r}}_{S}||}dS=\frac{q_{i}}{4\pi \varepsilon_{0}\varepsilon_{in}}\int \frac{\left({\vec{r}}_{i}-{\vec{r}}_{S}\right)\cdot \vec{n}\left({\vec{r}}_{S}\right)}{||{\vec{r}}_{i}-{\vec{r}}_{S}|{|}^{4}}\frac{dS}{4\pi }$$

The reaction field may be used to compute the Born radius according to equation (1) with *ε_out _*= ∞. The Coulomb field approximation for the grounded conducting surface has the advantage that the integral of the surface polarization charges will be always equal to the opposite of the charge inside the surface as for the exact solution. On the other hand it gives a very bad approximation both of polarization charges, which depend only on the distance from the source charge and on the normal to the surface, and reaction field energies even for simple systems. Although the Coulomb field approximation is reasonable, Grycuk has pointed out that it is fundamentally inaccurate for charges embedded in a sphere [30] by comparing both the self energy computed under the Coulomb field approximation and the pair interaction energy computed using the formula by Still and coworkers [15], with the exact solution obtained by using the Kirkwood model [6]. Based on the analysis for charges embedded in a sphere an exact formula is provided which may be recast as a function of a surface integral:

(6) $$U_{i}^{react}=-\frac{q_{i}}{4\pi \varepsilon_{0}\varepsilon_{in}}{\left(\int_{S}\frac{\left({\vec{r}}_{S}-{\vec{r}}_{i}\right)\cdot \vec{n}\left({\vec{r}}_{S}\right)}{||{\vec{r}}_{S}-{\vec{r}}_{i}|{|}^{6}}\frac{dS}{4\pi }\right)}^{\frac{1}{3}}$$

from which the integral expression for the generalized Born radius follows:

(7) $$\alpha_{i}=\frac{1}{{\left(\int_{S}\frac{\left({\vec{r}}_{\text{S}}-{\vec{r}}_{i}\right)\cdot \vec{n}\left({\vec{r}}_{\text{S}}\right)}{||\vec{r}\text{s}-{\vec{r}}_{i}|{|}^{6}}\frac{dS}{4\pi }\right)}^{\frac{1}{3}}}$$

The above expression which will be referred hereafter as GBR6 following Grycuk [30] and Tjong and Zhou [31,32], has been analysed in detail by Mongan et al. [33]. Equation 7 was found to perform extremely well also for "very" non spherical shapes and in the context of biomolecular models. Occasional large differences with respect to Poisson-Boltzmann calculations were found for inner cavities and local concavities at the surface, i.e. in conditions where a continuum model is anyway questionable. That study concluded that with a correction for a small systematic error, the GBR6 model is a sufficiently accurate continuum electrostatic model [33].

Tjong and Zhou [31,32] used an analytical implementation for the estimation of Born radii based on volume integrals (corresponding to the above formula (7) that uses a surface integral instead) and showed its superior accuracy compared to other existing methods for a set of 55 very different proteins. In their approach it is made clear that standard estimations of Born radii compute in fact only geometric properties, and they provide empirical formulae for the correct Born energy depending on the inner and outer dielectric constants, ionic strength, total charge and number of atoms.

Although successful and theoretically correct for a charge inside a grounded conducting sphere, there is no reason why equation (7) should provide good estimation of Born radii for complex shapes like those of proteins and biomolecules in general. In general Born radii could be estimated using integrals of the form (see the subsection Exact generalized Born radii for a conducting sphere):

(8) $$I_{n}=\int_{S}\frac{1}{4\pi }\frac{\left({\vec{r}}_{S}-{\vec{r}}_{i}\right)\cdot \vec{n}\left({\vec{r}}_{S}\right)}{||{\vec{r}}_{S}-{\vec{r}}_{i}|{|}^{n+1}}dS$$

In the absence of a theory which could be cast in a fast computational framework, fitting approaches have been successfully followed and tested on large sets of proteins showing excellent agreement with Poisson-Boltzmann calculation results. Romanov et al. [34] proposed that a linear combination of integrals *I*_3 _to *I*_6_, in the present notation, and a constant term could fit the self-polarization energy and thus be used to compute generalized Born radii (see also the discussion by Mongan et al. [33]).

### Applications of the generalized Born model

Based on the correct estimation of Born radii, the solvation contribution to the interaction between any two charges may be computed by using equation (2). Derivation of equation (2) with respect to atomic positions gives the electrostatic solvation forces acting on atoms. The implicit dependence of Born radii on atomic positions makes computation of forces far from trivial [35-40] for the generalized Born model as well as for the parent Poisson-Boltzmann model where the boundaries depend on atomic positions [41]. The possibility of computing energies and forces faster with respect to the reference Poisson-Boltzmann equation has made the Generalized Born model the choice of election for implicit solvent molecular dynamics simulations. Also, the computation of pairwise solvation energies allows for fast computation of pKa of multiple titrating groups as we discuss in the Methods section.

Applications of generalized Born model (and other implicit solvent methods) have been reviewed elsewhere [3,11-14]. At variance with the reference Poisson-Boltzmann model, the computation of electrostatic potential in space is outside the scope of the Generalized Born model where only interactions are considered thorugh equation 2. Here we use a finite radius test charge in order to define a potential within the frame of the generalized Born model.

### Aim of this work

In the present work we provide a software that, based on generalized Born radii, provides most common electrostatic analyses of proteins. The program first computes generalized Born radii, via surface integrals, based on different definitions and then it uses generalized Born radii (using a finite radius test particle, where needed) to perform electrostic analyses. In particular the ouput of the program entails, depending on user's requirement:

1) the generalized Born radius of each atom;

2) the solvation electrostatic free energy;

3) the electrostatic forces on each atom (the theory of electrostatic forces based on surface integrals is developed here and implementation is still at a developmental stage);

4) the pH-dependent properties (total charge and pH-dependent free energy of folding in the pH range -2 to 18);

5) the pKa of all ionizable groups;

6) the electrostatic potential at the surface of the molecule;

7) the electrostatic potential in a volume surrounding the molecule.

## Results and discussion

### Generalized Born radii

The Born radii were computed from numerical surface integrals and the resulting self energies were compared with the reference ones obtained from the Poisson-Boltzmann equation for 1000 atoms randomly chosen in the test set of 55 proteins. Born radii and Poisson-Boltzmann self-energies have been computed using the solvent accessible surface as dielectric boundary or the molecular surface computed using the program MSMS [42]. The surface points density used for MSMS computations was 10 pts Å^-2^. Table 1 reports the results concerning the self-energies obtained using different ways to estimate the generalized Born radii. It is seen that the GBR6 model performs very well and that the gain in accuracy when linear combinations of a constant term and self-energies corresponding to radii *α_n _*are used, with *n *ranging from 3 to 6 (LC5) and 3 to 10 (LC9), is limited.

**Table 1:** Self energies

model	RMSD	**corr. coef**.
GBR6 SAS	10.0	0.995

LC5 SAS	3.5	0.996

LC9 SAS	3.5	0.996

GBR6 MS	57.6	0.949

LC5 MS	23.9	0.950

LC9 MS	22.7	0.955

Comparison of self-energies computed using generalized Born radii from surface integrals and using the Poisson-Boltzmann equation. For each computational model (SAS stands for solvent accessible surface, MS stands for molecular surface, see text) the average root mean square difference (RMSD) (kJ/(q mol)) and the correlation coefficient are reported.

**Figure 1:**
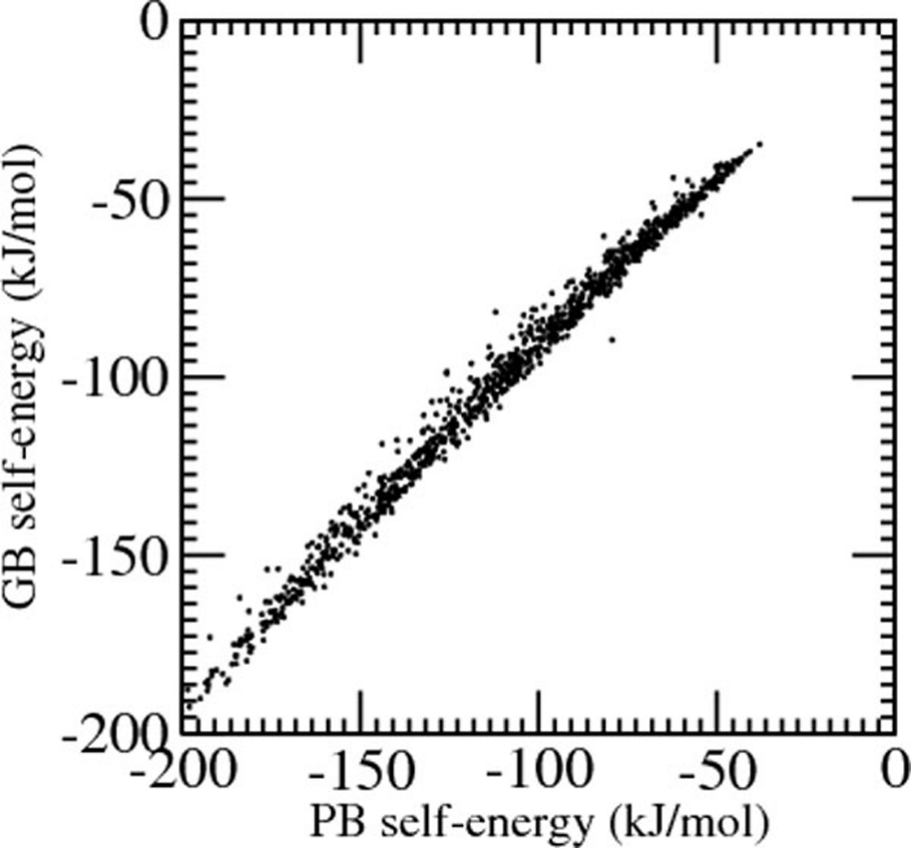
**GB vs. PB self energies**. Generalized Born self-energies using solvent accessible surface integrals versus Poisson-Boltzmann self-energies computed using the program APBS.

For best performance it is necessary to use a number of probe points per atom larger than 100. For less dense surface points occasional negative sign integrals are observed which need an adhoc treatment, e.g. resetting the generalized Born radius to a predetermined value. Although negative radii are not found for higher densities of surface points, there are still rarely occurring large deviations between GB and PB self-energies, which could be due to differences in the computation of the boundary surface and its further treatment in the two approaches. As an illustration of the accuracy obtained, Figure 1 reports the plot of generalized Born self-energies versus those computed using APBS.

### Electrostatic solvation free energy

**Figure 2:**
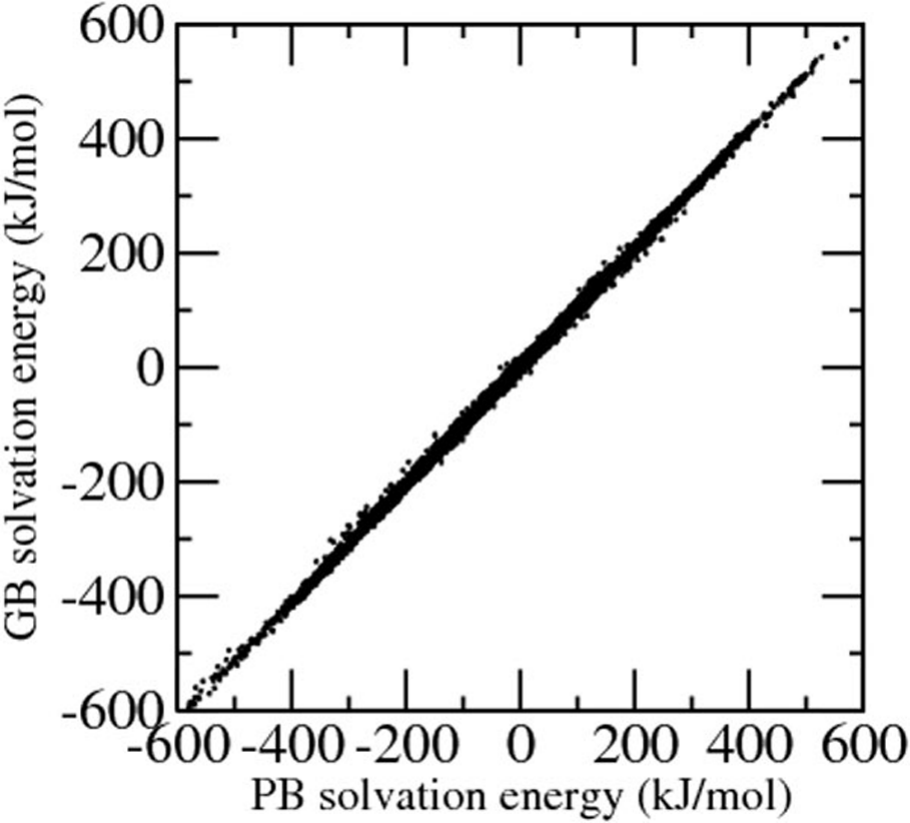
**GB vs. PB solvation energies**. Generalized Born solvation energies at each atom using solvent accessible surface integrals versus the corresponding Poisson-Boltzmann energies computed using the program APBS.

The electrostatic solvation energy computed using the solvent accessible surface integral (model GBR6) at each atom, computed as half of the product charge times reaction field, was compared with the corresponding solvation energy computed using the Poisson-Boltzmann equation. Compared to the solvation self-energy, the solvation energy depends also on other charges in the molecule and provides a test for the solvation effects computed according to equation 2. The average root mean square deviation from reference Poisson-Boltzmann calculations is just 2.3 kJ/mol and the correlation coefficient is 0.9996. The accuracy of the GB model versus the Poisson-Boltzmann model may be judged from Figure 2 which reports the solvation energy (computed as half the product of charge times reaction field) at each atom for the 93240 atoms of the 55 protein set.

### Electrostatic solvation forces

Since solvation energies depend, according to equation 2, on atomic positions explicitly and implicitly through Born radii, the computation of solvation electrostatic forces is quite complex, and strongly dependent on the interface model chosen [38-40] (see also for a general discussion [41]).

In order to estimate how important are effects due to the dependence of Born radii on atomic positions, solvation electrostatic forces have been computed for each atom of the 55 proteins as the derivative of the solvation energy under the approximation that Born radii are constant. Albeit approximate this way of computing electrostatic forces preserve by definition zero total electrostatic force as expected for isotropic media and as found by the correct expression for the force [41]. Due to the different way of computing forces we did not attempt comparing ionic boundary, dielectric boundary and charge times electrostatic field components of the electrostatic force, but we rather compared the total solvation force. The results are not very accurate with the average root mean square deviation, with respect to the forces computed using APBS using the same parameters, equal to 2.7 kJ/(Åmol) compared to an average square root value of the force of 5.0 kJ/(Åmol). The correlation coefficient is 0.84.

**Figure 3:**
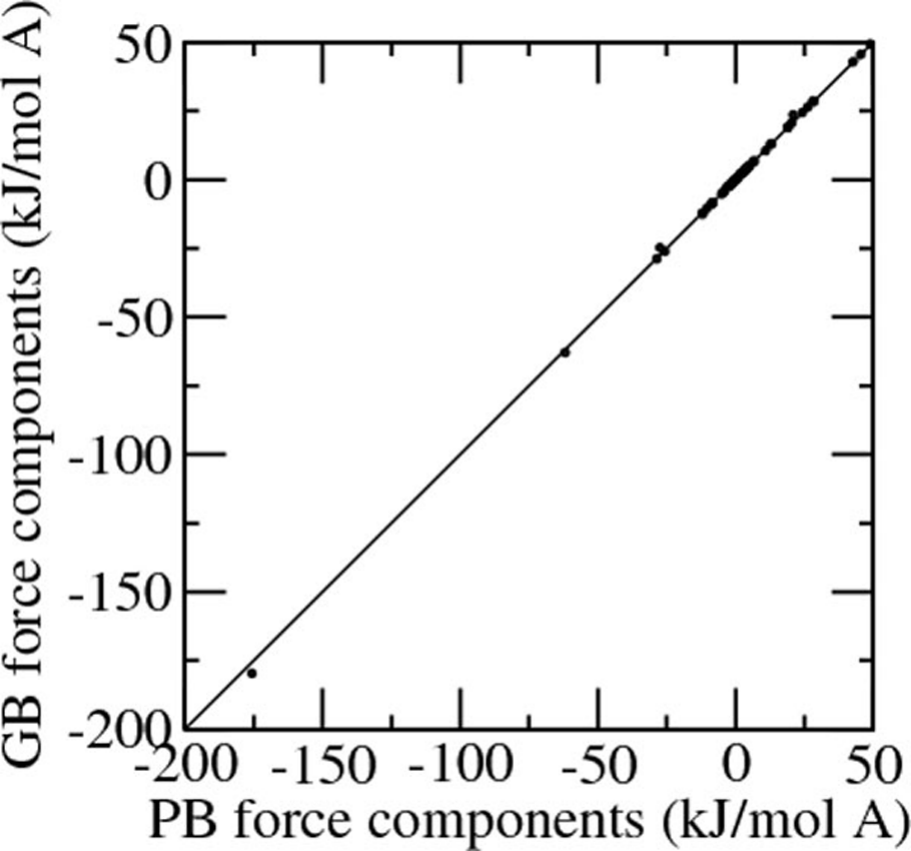
**GB vs. PB forces**. Generalized Born force components at 20 random points within a 5.0 Å sphere with random charges, computed using solvent accessible surface integrals versus the corresponding Poisson-Boltzmann energies computed using the program APBS.

The full computation of electrostatic solvation forces using surface integrals is described in the Methods section. As a demonstration of the accuracy of the calculation 20 random point charges ranging between -1.0 and 1.0 q have been embedded in a sphere of radius 5.0 Å at a distance of at least 0.5 Å from each other. Besides small differences due to treatment of the boundary, the agreement between force components computed using surface integrals and those computed solving the Poisson-Boltzmann equation is very good, with a correlation coefficient of 0.9998 and a fitting slope of 0.983 (Figure 3). Work is under way to implement force calculation in an efficient way for large systems like proteins.

### pH-dependent properties (total charge, pH-dependent free energy of folding, and pKa of ionizable groups

The test set provided by Gunner and coworkers has been used to test the prediction of pKa's in proteins [43]. We compared the results obtained with the results obtained by running the program Propka2.0 [44-47]. The latter program is very fast and provides predictions whose accuracy is comparable to that of more computationally intensive programs. We chose this program as a reference because it is widely used and it is seemingly the fastest available with a very good accuracy. A wide range of programs and methods have been compared recently [48] and the reader is referred to that work for more extensive comparisons of existing softwares.

The results are summarized in Table 2. It should be noted that the method used here was not extensively optimized to maximize correlation with experimental results or to minimize the root mean square deviation (RMSD) with respect to the experimental data.

**Table 2:** Predicted vs. experimental pKa shifts

	BLUUES		Propka v. 2.0	
	**RMSD**	**corr. coef**.	**RMSD**	**corr. coef**.

ASP	0.78	0.42	1.50	0.27

GLU	0.90	0.54	1.75	0.24

HIS	0.98	0.74	1.50	0.59

LYS	0.79	0.45	0.79	0.25

TYR	1.78	0.41	2.12	-0.03

NTR	0.54	0.93	0.96	-1.00

CTR	1.01	-0.14	1.06	0.36

All	1.00	0.51	1.59	0.36

Comparison of predicted vs. experimental pKa's. The RMSD is reported together with the correlation coefficient. We report the results obtained using Propka v.2.0. The latter program is very accurate, but occasional large shifts, not filtered nor treated ad-hoc here, result in large RMSD and smaller correlation coefficients.

On the other hand some scaling of contributions is done and therefore large deviations from experimental results are not found. In this respect, Propka that uses ten adjustable parameters and other parameters chosen for best performance [46] apparently does not prevent very large shifts to be predicted. As a consequence the global RMSD from experimental data is large and could thus easily be reduced. The execution time of Propka is in the range of seconds, while our program runs in minutes. No optimization or approximation has been implemented as yet.

**Figure 4:**
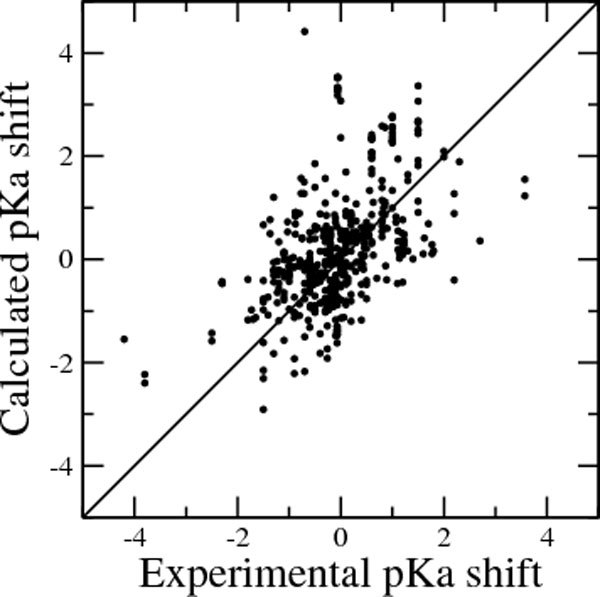
**GB vs. PB pKa shifts**. Computed versus experimental pKa shifts (compared to the reference model ones) for the dataset of Gunner and coworkers [43].

The results are somewhat better than those obtained by Propka (v. 2.0), although the figures reported here for the performance of Propka (v. 2.0) are worse than those reported in the original papers, because of a different test set. We remark that the comparison reported in Table 2 is dominated by outliers, that could be easily filtered or treated in an ad hoc manner, for Propka. Similar figures are found in independent tests [48-50]. An additional problem is that the data set includes multimeric (up to decameric) proteins and therefore the latter contribute more than others to the figures reported in Table 2. It should be noted that this comparison is far from exhaustive as many other features could be chosen to judge a method's value. Extensive comparisons have been performed by Stanton and Houk [48]. The purpose of the comparison performed here is to demonstrate that our program outputs results comparable (or better on the dataset used here) than the results obtained by one of the best state-of-the-art method. In order to better judge the performance of the method, computed versus experimental shifts in pKa values, with respect to the reference model ones, are reported in Figure 4.

### Surface electrostatic potential

The evaluation of the potential in regions outside the molecule is beyond the scope of the generalized Born approach which focuses instead on self and interaction energies. This is at variance with approaches that approximate the potential computed using the Poisson-Boltzmann equation. In particular Onufriev and coworkers [7,8] have found an approximation (not simply amounting to a truncation) based on Kirkwood's series expansion of the solution for a sphere [6]. Their analytical model performs surprisingly well for a large set of proteins and enables fast calculation of the potential at the surface and in the volume surrounding the molecule.

**Figure 5:**
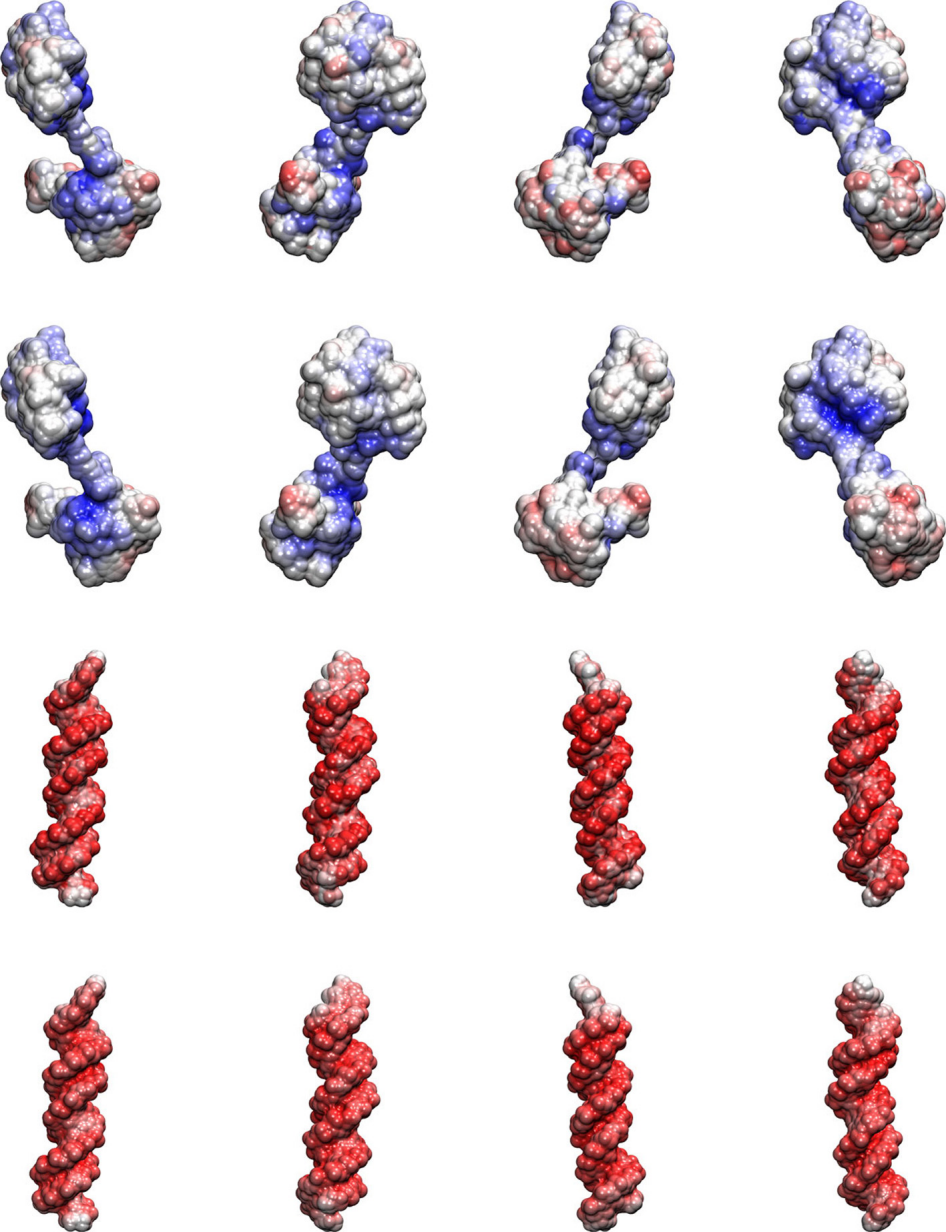
**GB vs. PB surface potential (average over atomic surface)**. Computed surface potential espressed in kcal/(mol q) for the paired domain of the protein PAX6 and its cognate DNA (PDB structure id.: 6PAX). The reference Poisson-Boltzmann potential (lower row) and the average generalized Born potential at atomic surface (upper row) are displayed with colors ranging from red (-3.0 kcal/(mol q)) to blue (3.0 kcal/(mol q)) for DNA and from red (-2.4 kcal/(mol q)) to blue (2.4 kcal/(mol q)) for the protein.

The surface potential has been computed here, within the framework of generalized Born approach, as the energy of interaction of all atoms of the molecule with a unit test charge with a generalized Born radius equal to half the solvent probe radius, i.e. 0.7 Å. This value was found to reproduce well the potentials computed using APBS. Each surface point is assigned to the atom contacted by the solvent and for each solvent exposed atom the average surface potential is listed in the beta factor field in the output pdb file. The result is thus a readable list of atoms with the corresponding average surface potential if the atom is solvent exposed. This information may be used to display the potential using softwares like VMD [51]. As an example the potential computed using the Poisson-Boltzmann equation and using the method described above is shown in Figure 5 for the paired domain of the protein PAX6 and its cognate DNA. Exactly the same parameters are used in the two calculation with the only difference that the surface in the Poisson-Boltzmann calculation is generated using van der Waals radii inflated by 0.7 Å. The boundary surfaces are however different in the two calculations and this value was found to compensate for the differences.

**Figure 6:**
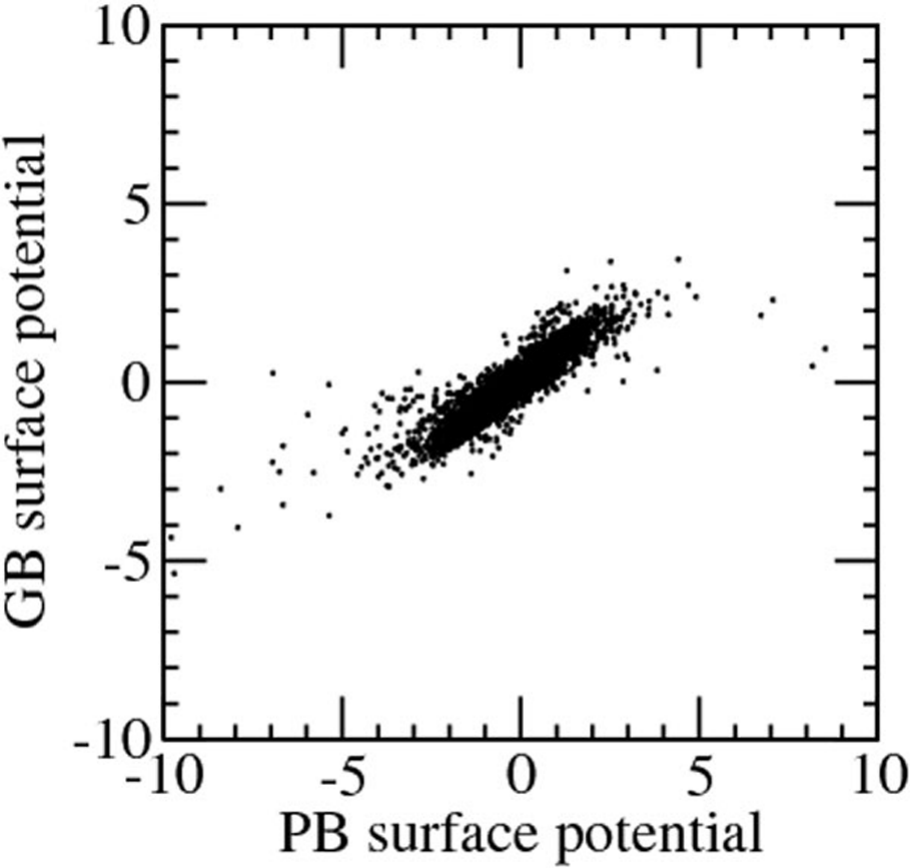
**GB vs. PB surface potential**. Surface potential, espressed in kcal/(mol q), computed using the generalized Born model, versus the reference Poisson-Boltzmann potential at the same surface point.

For the same reason a point by point comparison of the potential at the surface is not completely appropriate, because boundaries are slightly different in the two calculations. Instead of taking the average surface atomic potential, we compared the potential computed using the generalized Born model and a 0.7 Å radius test charge at each surface point with the Poisson-Boltzmann potential computed by UHBD at the same point. Such comparison is reported in Figure 6 and, notwithstanding the differences in methodologies, the correlation coefficient is 0.90 and the average error is just +0.08 kcal/(mol q). The root mean square deviation is 0.43 kcal/(mol q) with the largest contributions to this figure due to outliers by several standard deviations. Outliers are found in inner cavities, but also at concavities at the exposed surface. In some cases the errors are due to the differences in boundary definition. The error distribution is however similar to that reported by Onufriev and coworkers [8].

### Electrostatic potential in a volume surrounding the molecule

**Figure 7:**
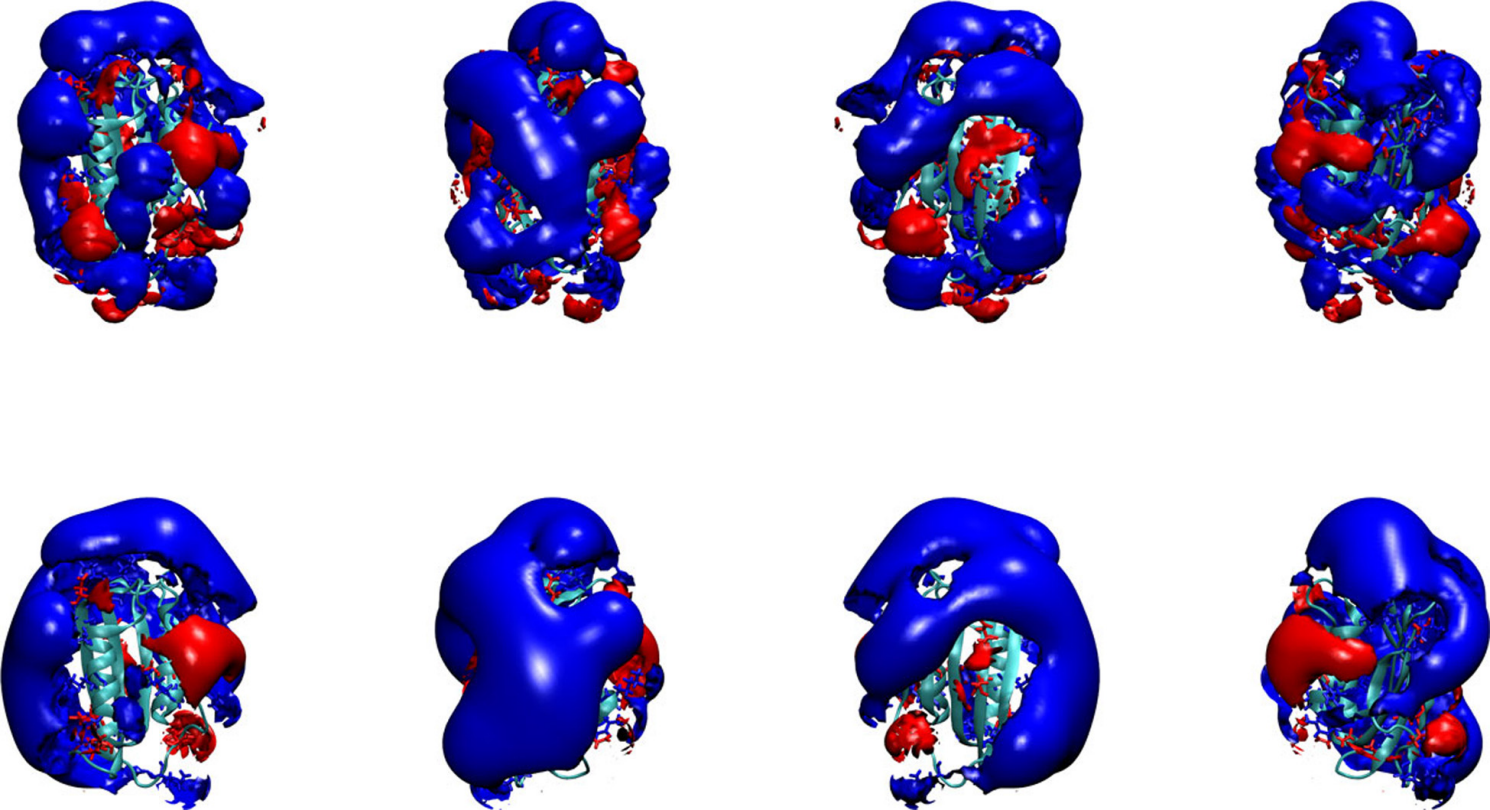
**GB vs PB isopotential curves**. Isopotential curves at 0.3 kcal/(mol.q). Upper row: potential obtained using the GBR6 model; lower row: potential obtained by solving the Poisson-Boltzmann equation.

Similar to the surface potential the electrostatic potential for a unit test charge with generalized Born radius equal to half of the solvent probe radius may be computed at nodes of a grid enclosing the molecule and output in the DX format (the DX file format is described at [52]) readable by programs like VMD [51]. The grid is chosen based on as the minimum box entailing the molecule plus three Debye length on each side. If the Debye length is more than 10 Å, 30 Å are added at the minimum box on each side. The space is then divided in 97 × 97 × 97 grid points and the potential is computed at each point. The method is thus slower compared to solving the finite difference equation for the potential. At variance with standard options with other software, here the potential inside the molecule is set to 0, which removes isopotential curves inside the molecules (uninformative because arising from strong varying local fields) and helps with the visualization of the structure of the molecule together with outer isopotential curves. Example input files are given in the supplementary material. The comparison with the analogous analysis using the program APBS shows less smooth isopotential surfaces (Figure 7), a fact which might be due to the finite radius of the unit charge test particle. Note that the output potential is in kJ/(q mol) compared to widely used kT/(q mol) or kcal/(q mol).

## Conclusions

A program for the analysis of the electrostatic properties of proteins based on the surface integral computation of generalized Born radii has been presented. Further work will be devoted to improve the efficiency of calculations in particular for what concerns electrostatic solvation forces. The program, together with examples is given as supplementary material (Additional file 1).

## Methods

### Reference APBS and UHBD calculations and parameters

Reference electrostatic calculations were performed with the programs APBS [53] and UHBD [54,55]. Except where noted, standard parameters were used: inner dielectric constant 1.0, outer dielectric constant 78.54, temperature 298.15 K, ionic strength 0.150 M. In APBS the mesh of the grid was 0.25 Å which resulted in very large grid size (257^3 ^points). In UHBD, used for surface potential calculations, a focusing procedure was used with the final mesh size of 0.8 Å.

The boundary surface was defined in the two programs in order to match as close as possible the definition used in our program, i.e. the molecular surface for atoms with radii inflated by the radius of the solvent. The surface point density was 10 Å^-2^.

Except where noted the same temperature, dielectric constant and ionic strength were used in our program.

### Solvent accessible surface

Solvent accessible surface points and surface normal vectors have been generated by considering the van der Waals sphere of each atom inflated by the radius of the solvent (e.g., for an atom of radius 1.9Å and a solvent radius of 1.4Å the inflated radius was 3.3Å).

Points on the inflated van der Waals sphere and surface normal vectors are precomputed for a set of inflated radii (*r*) and centered at atom positions. The coordinates of the *i*-th point (*i *ranging from 1 to *n_pt_*) on the sphere are generated according to the following rule based on the golden section (due to Anton Sherwood, see http://www.cgafaq.info/wiki/Evenly_distributed_points_on_sphere and links thereof):

$$\begin{array}{c} z_{i}=r\times \left(1-2\frac{i-1+\frac{1}{2}}{n_{pt}}\right)\\ x_{i}=\sqrt{r^{2}-z_{i}^{2}}\times \cos\left(\left(3-\sqrt{5}\right)\pi \left(i-1\right)\right)\\ y_{i}=\sqrt{r^{2}-z_{i}^{2}}\times \sin\left(\left(3-\sqrt{5}\right)\pi \left(i-1\right)\right) \end{array}$$

*n_pt _*was 200 corresponding for an atom with radius 1.9Å to a density of 4.4 pts Å^-2^.

Atoms are mapped on a grid and the position of each surface point is compared only with the position of the list of atoms associated with neighboring grid points. This procedure was found to be efficient and robust without any failure.

Each surface point within the inflated van der Waals volume of a different atom is considered buried. All surface integrals have been performed as the corresponding finite sums over exposed (non buried) surface points.

### Generalized Born radii

Reference Born radii have been obtained from Poisson-Boltzmann self-polarization energies computed using APBS [53] according to eq. 1.

Different Born radii have been obtained from surface integrals as:

$$\alpha_{n}=\sqrt[{n-2}]{\frac{1}{I_{n}}}$$

where *I_n _*is defined by equation 8, or by fitting the reference solvation self energy by linear combinations of the solvation energies corresponding to different *α_n_*'s (*n *ranging here from 3 to 6) as done by Romanov et al. [34]. Fitting was necessary because the published coefficients did not provide good results, as a result of the different procedures used to compute surfaces and solvation free energies.

### An equation for the generalized Born radius

The reaction field obeys Laplace equation in the solute region. When the reaction field is expressed via a generalized Born equation like (2), we may conveniently study what are the requirements imposed by Laplace equation on Born radii. In particular we consider the reaction field due to a source charge at ${\vec{r}}_{i}$ and consider the reaction field at a point ${\vec{r}}_{j}$, *U_ij_*, in the limit ${\vec{r}}_{j}\to {\vec{r}}_{i}$. Under this limit the reaction field may be approximated as:

(9) $$U_{ij}=\frac{-q_{i}}{4\pi \varepsilon_{0}\varepsilon_{in}\sqrt{r_{ij}^{2}K+\alpha_{i}\alpha_{j}}}$$

where the constant *K *depends on the coefficient used in the exponential and is 0.75 for the commonly adopted Still formula.

Imposing that ∇^2^*U_ij _*= 0, where all derivatives are with respect to coordinates of ${\vec{r}}_{j}$, and letting ${\vec{r}}_{j}$ tend to ${\vec{r}}_{i}$, after some straightforward manipulation, results in the following equation for the Born radius *α_i_*:

(10) $$\alpha \nabla^{2}\alpha -\frac{3}{2}\left(\vec{\nabla }\alpha \right)\cdot \left(\vec{\nabla }\alpha \right)+6K=0$$

For the conducting grounded surface approximation the boundary condition is that the Born radius approaches 0 as the boundary surface is approached. It is easy to check that this equation is satisfied for the sphere and the plane where *K *is equal 1.

For two parallel conducting infinite planes (approximated here by two very large circular plates) the solution may be compared with that obtained by expanding the exact solution in Bessel functions. We set the interplates distance along the *z*-axis equal to 1.0 and the radius *R *of the plates equal 1000.0. Consider *x *as the distance of the source charge from one of the plates on the axis of symmetry. The reaction field between the plates may be expressed as a sum of functions which satisfy the Laplace's equation in cylindrical coordinates:

(11) $$U_{react}\left(\rho ,z\right)=\underset{k}{\sum }\left(c_{k}^{+}e^{kz}+c_{k}^{-}e^{-kz}\right)J_{0}\left(k\rho \right)$$

where *J*_0_(*x*) is the Bessel function of order zero. We may consider only order 0 functions due to cylindrical symmetry. The coefficients are obtained by imposing that at the boundary the potential (*U_react _*+ *U_source_*) is equal to zero and using the orthogonality relation: $\int xJ_{0}\left(kx\right)J_{0}\left(k^{\prime }x\right)=\frac{1}{k}\delta \left(k-k^{\prime }\right)$. The parameters k are chosen such that *J*_0_(*kR*) = 0.

The solution of equation (10) is obtained by bisection, guessing first a value at the midpoint of the axis of symmetry and integrating the equation using an adaptive Runge-Kutta fourth order method [56] and requiring that the value of the Born radius at the boundary be 0.

**Figure 8:**
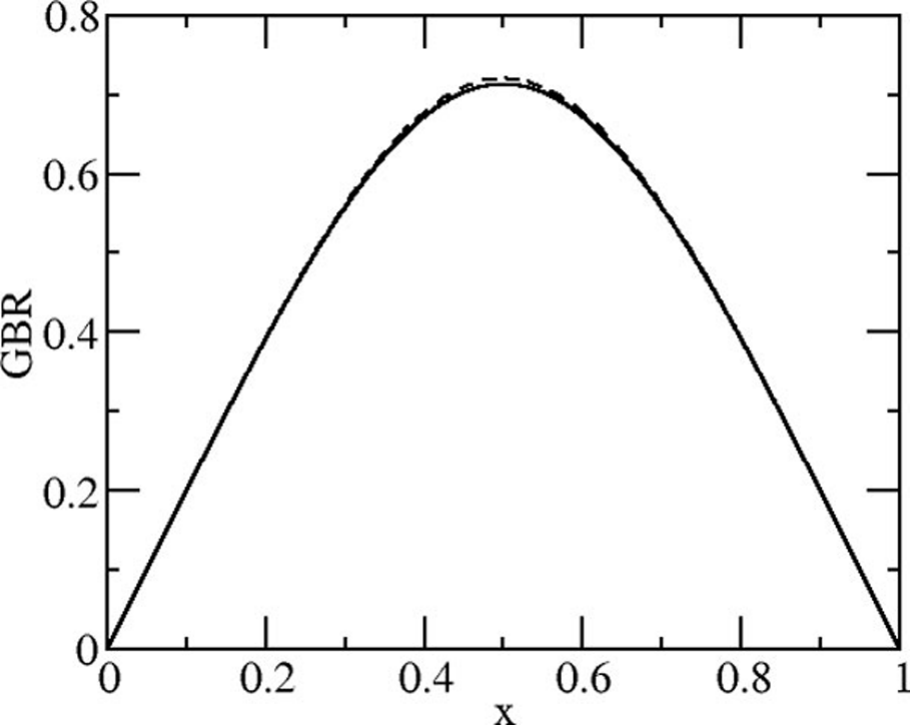
**GB radius from Equation (10) vs. numerical solution**. Computed generalized Born radius for a charge between two infinite planes depending on the distance *x *from one of the planes. The generalized Born radius and the distance are normalized by the plane to plane distance. Solid line refers to the solution obtained from equation (10) and dashed line refers to the expansion (11) of the solution for a finite system.

The two solutions reasonably agree, within numerical accuracy, and are compared in Figure 8. Solving Equation (10) for proteins could represent an alternative to other well established methods to compute generalized Born radii. Obviously all the above derivation rests on the assumption that Still's formula (or alike) provides a good approximation of the exact solution to the Poisson (or Poisson-Boltzmann) equation for non-homogeneous media.

### Electrostatic solvation energies in ionic solutions

A merit of generalized Born models is to separate Coulombic interactions from reaction field effects. The generalized Born radii have been used to compute interactions in ionic solutions assuming that charges whose distance is much larger than their Born radii interact through a Debye-Huckel potential and that the self-polarization energy can be computed according to the Debye-Hückel law [57]. The expression used in the present work for the reaction potential due to a unit source charge *i *at the site *j *for an ionic solution of dielectric constant *ε_out _*and Debye constant *k_D _*is:

(12) $$U_{i,j}^{solv}=-\frac{1}{4\pi \varepsilon_{0}\sqrt{r_{ij}^{2}+\alpha_{i}\alpha_{j}e^{\frac{-r_{ij}^{2}}{4\alpha_{i}\alpha_{j}}}}}\left(\frac{1}{\varepsilon_{in}}-\frac{e^{-k_{D}}\sqrt{r_{lj}^{2}+\alpha_{i}\alpha_{j}e^{\frac{-r_{ij}^{2}}{4\alpha_{i}\alpha_{j}}}}}{\varepsilon_{out}e^{-k_{D\sqrt{\alpha_{il}\alpha_{j}}}}\left(1+k_{D}\sqrt{\alpha_{i}\alpha_{j}}\right)}\right)$$

which is slightly different from that reported by Srinivasan et al. [57] in order to include dielectric constant different from 1. When we fit the self-polarization energy obtained by the above equation versus that obtained by equation (22) in the work Tjong and Zhou [31], using a zero intercept line and assuming a physiological ionic strength of 0.150 M we find a correlation coefficient of 0.999 and a slope of 0.999. The total solvation energy is obtained by summing contributions from all pairs of atoms and solvation self-energies:

(13) $$G_{solv}=\frac{1}{2}\underset{i,j=1,N}{\sum }q_{i}q_{j}U_{i,j}^{solv}$$

The total energy of the molecule, which does not include self-energies in the inner dielectric medium is:

(14) $$G_{tot}=\frac{1}{2}\underset{i,j=1,N}{\sum }q_{i}q_{j}\phi_{i,j}=\frac{1}{2}\underset{i,j=1,N}{\sum }q_{i}q_{j}\left(\frac{1-\delta_{i,j}}{4\pi \varepsilon_{0}\varepsilon_{in}r_{ij}}+U_{i,j}^{solv}\right)$$

where the functions *ϕ*_*i*,*j *_are the Green's functions at points *i *and *j *but do not include the self potential of each charge in the inner dielectric medium and *δ*_*i*,*j *_is Kronecker's delta.

### Electrostatic solvation forces

The electrostatic solvation forces are obtained by differentiation of *G_solv _*with respect to atomic coordinates. The implicit dependence of Born radii on atomic coordinates through a surface integral makes the derivation rather tedious. One approximation is to consider that effects from the variation of Born radii with atomic coordinates are smaller than the explicit variation of energies due to the change in interatomic distances. This is in general not the case. Just consider the force arising from variation of the self-energy with atomic coordinates. For a point charge *q *in a conducting sphere of radius *R *at a distance *a *from its center the solvation force is pointing away from the center of the sphere and its magnitude is:

(15) $$F=\frac{q^{2}}{4\pi \varepsilon_{in}}\frac{aR}{{\left(R^{2}-a^{2}\right)}^{2}}$$

For a unit charge placed at 7.5 Å from the center of a globular protein of radius 10 Å, assuming an inner dielectric constant 4.0, the resulting force is 14 kJ/(mol Å). An opposite force of the same magnitude is applied on the center of the sphere defining the boundaries. This simple example shows that forces arising from variations of Born radii are not negligible.

The derivative of electrostatic potential implies derivatives of Born radii with respect to atomic coordinates that may be computed as derivatives of a function of a surface integral:

(16) $$\frac{d\alpha_{i}}{dx_{k}}=\frac{d{\left(\frac{1}{I_{5}^{i}}\right)}^{\frac{1}{3}}}{dx_{k}}=-\frac{1}{3}{\left(\alpha_{i}\right)}^{4}\frac{dI_{5}^{i}}{dx_{k}}$$

The derivative of $I_{5}^{i}$ is not straightforward as the domain of integration depends on atomic coordinates.

The subtleties of derivation of integrals have been described by Flanders [58]. We use here a form valid for the derivation of a flux through a surface that does not entail the derivative of the boundary. The total derivative with respect to a variable *t *(typically time, here atomic coordinates) of the flux of a vector field $\vec{F}$ through a surface *S *is expressed as a surface integral [59]:

(17) $$\frac{d}{dt}\int_{S}\vec{F}\cdot \vec{n}\left({\vec{r}}_{S}\right)dS=\int_{S}\left[\frac{\partial \vec{F}}{\partial t}+\left(\vec{\nabla }\cdot \vec{F}\right)\vec{v}\left({\vec{r}}_{S}\right)+\vec{\nabla }\times \left(\vec{F}\times \vec{v}\left({\vec{r}}_{S}\right)\right)\right]\cdot \vec{n}\left({\vec{r}}_{S}\right)dS$$

where

$$\vec{v}\left({\vec{r}}_{S}\right)=\frac{d{\vec{r}}_{S}}{dt}$$

The surface point (for the solvent accessible surface) is dependent on the contacting atom (say the *j^th ^*atom):

(18) $${\vec{r}}_{S}={\vec{r}}_{j}+\vec{n}\left({\vec{r}}_{S}\right)\left(r_{vdW,j}+r_{p}\right)$$

where *r*_*vdW*,*j *_is the van der Waals radius of atom *j *and *r_p _*is the radius of the solvent. The derivative of ${\vec{r}}_{S}$ with respect to the *k^th ^*coordinate of atom *l *takes a very simple form:

(19) $$\frac{d{\vec{r}}_{S}}{dx_{l,k}}=\delta_{l,j}{ê}_{k}$$

where ${ê}_{k}$ is the versor of the *k^th ^*axis. Further derivations of ${\vec{r}}_{S}$ will be zero. Taking into account the latter statement and expanding the third term in the right-hand side of equation 17 a simpler form of the same equation is found:

(20) $$\frac{d}{dt}\int_{S}\vec{F}\cdot \vec{n}\left({\vec{r}}_{S}\right)dS=\int_{S}\left[\frac{\partial \vec{F}}{\partial t}+\left(\vec{v}\left({\vec{r}}_{S}\right)\cdot \vec{\nabla }\right)\vec{F}\right]\cdot \vec{n}\left({\vec{r}}_{S}\right)dS$$

The left-hand side of equation 20 is directly related to equation 16 with:

$$\vec{F}=\frac{1}{4\pi }\frac{{\vec{r}}_{i}-{\vec{r}}_{S}}{||{\vec{r}}_{i}-{\vec{r}}_{S}|{|}^{6}}$$

We define a function $is\;\left({\vec{r}}_{S}\right)$ that assigns to each surface element the index of the atom defining the boundary and consider the derivative of $I_{5}^{i}$ with respect to the *k^th ^*coordinate of atom *l*:

(21) $$\frac{dI_{5}^{i}}{dx_{l,k}}=\int_{S}\left[\delta_{l,i}\frac{\partial }{\partial x_{l,k}}\left(\frac{1}{4\pi }\frac{\left({\vec{r}}_{i}-{\vec{r}}_{S}\right)}{||{\vec{r}}_{i}-{\vec{r}}_{S}|{|}^{6}}\right)+\delta_{l,is\left({\vec{r}}_{\text{S}}\right)}\left(\frac{d{\vec{r}}_{S}}{dx_{l,k}}\cdot \vec{\nabla }\right)\left(\frac{1}{4\pi }\frac{\left({\vec{r}}_{i}-{\vec{r}}_{S}\right)}{||{\vec{r}}_{i}-{\vec{r}}_{S}|{|}^{6}}\right)\right]\cdot n\left({\vec{r}}_{S}\right)dS$$

(22) $$=\int_{S}\delta_{i,l}\left(\frac{n_{k}\left({\vec{r}}_{S}\right)}{4\pi ||{\vec{r}}_{i}-{\vec{r}}_{S}|{|}^{6}}-\frac{6}{4\pi }\frac{\left({\vec{r}}_{i}-{\vec{r}}_{S}\right)\cdot n\left({\vec{r}}_{S}\right)}{||{\vec{r}}_{i}-{\vec{r}}_{S}|{|}^{8}}{\left({\vec{r}}_{i}-{\vec{r}}_{S}\right)}_{k}\right)dS$$

(23) + implicit surface-dependent terms

Note that in the above equations the operator $\vec{\nabla }$ operates only on *r_S _*coordinates, i.e. at the point where the integrand function is evaluated.

Let us consider now a simplified form (exact for a conducting sphere) of the Green's function for the electrostatic solvation energy

(24) $$U_{i,j}=-\frac{1}{4\pi \varepsilon_{0}\varepsilon_{in}\sqrt{r_{ij}^{2}+\alpha_{i}\alpha_{j}}}$$

and calculate explicitly the solvation force along the *k^th ^*coordinate of atom *j*:

(25) $$f_{l,k}=-\frac{dG_{solv}}{dx_{l,k}}=-\frac{d\left(\frac{1}{2}\sum_{i,j}q_{i}q_{j}U_{i,j}\right)}{dx_{l,k}}$$

(26) $$=\frac{1}{4\pi \varepsilon_{0}\varepsilon_{in}}\underset{j}{\sum }q_{l}q_{j}\frac{\left(x_{l,k}-x_{j,k}\right)}{{\left(\sqrt{r_{lj}^{2}+\alpha_{i}\alpha_{j}}\right)}^{3}}$$

(27) $$+\frac{1}{12}\frac{1}{4\pi \varepsilon_{0}\varepsilon_{in}}\frac{\alpha_{i}\alpha_{j}}{{\left(\sqrt{r_{lj}^{2}+\alpha_{i}\alpha_{j}}\right)}^{3}}\left(\alpha_{i}^{3}\frac{dI_{5}^{i}}{dx_{l,k}}+\alpha_{j}^{3}\frac{dI_{5}^{j}}{dx_{l,k}}\right)$$

where the apices *i *and *j *in *I*_5 _means that the integral involves the vector from the surface points to the coordinates of atoms *i *and *j *respectively. The computation of forces via surface integral according to the above equations is not practical, unless a cutoff on the distance between pairs of atoms and surface points is used. Indeed for each of the $\frac{N\left(N+1\right)}{2}$ pairs of atoms *j *and *i*, where *N *is the number of atoms, surface integrals must be computed (see equation (21)). This is the result of the brute force application of our surface integral definition of Born radius. Work is under way in our laboratory to make this computation efficient.

### pH-dependent properties (total charge and pH-dependent free energy of folding in the pH range -2 to 18)

The method described by Antosiewicz et al. [60] is implemented here with some modifications. PDB entries corresponding to proteins for which data are available in the pKa database made available by Gunner and coworkers [43] were prepared with pdb2pqr [61].

The method implemented here is a single conformer method, at variance with other methods like the QM/MM/TI method by Simonson et al. [62], MCCE [43] and the constant pH molecular dynamics method by McCammon and coworkers [63]. These methods are expected to be more accurate, at the expense of large computational time.

Approaches similar to that implemented here have been used by Onufriev and coworkers [64,65] where a significant speedup for larger proteins, with respect to our implementation, is due to clustering of interacting sites.

Significant improvements over continuum electrostatics (and more in general molecular mechanics) methods have been achieved by parametrization of interactions [49,50,66-69].

Other approaches which use a continuum method have been proposed recently which achieve very good agreement with experimental values, by using a pentapetide reference model for the titratable group, and by optimizing the hydrogen positions [70].

Improvements similar to those mentioned in the above paragraphs will be implemented in the future in our program.

We summarize here the theory that is discussed at length by Antosiewicz et al. [60].

#### Energy of ionization

The free energy of ionization of an ionizable group in an unfolded polypeptide at a given pH is:

(28) $$\Delta G=\log\left(10\right)RTz\left(pK_{a}-pH\right)$$

where *z *is the charge of the group upon ionization, R is the gas constant, T is the temperature (298.15 K in the present calculations).

It is assumed that pKa of protein ionizable groups in an unfolded protein are the same as those of model compound. We assume here that the the pKa are the following: 4.5 for Glu, 3.8 for Asp, 6.5 for His, 12.5 for Arg, 10.5 for Lys, 9.0 for Tyr, 8.0 for Cys, 8.0 for the N-terminal ammine and 3.2 for the C-terminal carboxyl.

The protein environment may change the free energy of ionization. If we assume that the main effects are electrostatic in nature the free energy of ionization for groups in a folded proteins is:

(29) $$\Delta G=\underset{i}{\sum }\log\left(10\right)RTz_{i}\left(pK_{a}^{i,M}-pH\right)$$

(30) $$+\frac{1}{2}\underset{ij}{\sum }\left(\left(q_{i}+z_{i}\right)\left(q_{j}+z_{j}\right)-q_{i}q_{j}\right)\phi_{i,j}^{N}$$

(31) $$-\frac{1}{2}\underset{ij}{\sum }\left(\left(q_{i}+z_{i}\right)\left(q_{j}+z_{j}\right)-q_{i}q_{j}\right)\phi_{i,j}^{M}$$

where, for simplicity of notation, the sum runs on all atoms of the protein. *q_i _*are the atomic charges in the neutral amino acids and *z_i _*is 0 for most atoms and 1 or -1 for those representing basic and acid ionized groups, repectively. Superscript *M *stands for the model compound representing the unfolded state and *N *stands for the natively folded protein. The model compound of ionizable residues is obtained by excising that residue from the protein. The Green's functions *ϕ*_*i*,*j *_are given by equation 14. The equation can be rearranged as follows:

(32) $$\Delta G=\underset{i}{\sum }\log\left(10\right)RTz_{i}\left(pK_{a}^{i,M}-pH\right)+\frac{1}{2}\underset{i}{\sum }z_{i}^{2}\left(\phi_{i,i}^{N}-\phi_{i,i}^{M}\right)+\underset{ij}{\sum }z_{i}q_{j}\left(\phi_{i,j}^{N}-\phi_{i,j}^{M}\right)+\underset{i>j}{\sum }z_{i}z_{j}\phi_{i,j}^{N}$$

where each term describes a contribution to the free energy of ionization: the first term is the free energy of ionization in the model compound, the second term is the difference in ionization self-energy, the third term describes the difference in interaction of ionization charges with partial charges in the unionized protein and model compound and the fourth term describes the interaction among ionization charges. Following Antosiewicz et al. the inner dielectric constant is set to 20.0. A wide range of inner dielectric constants has been used for pKa calculations. When a single dielectric constant has been used it has been chosen mostly larger than 1, e.g. 4 in MCCE [43], 8 in EGAD [68] and 11 in GB/IMC [70]. A large value of the dielectric constant accounts in an empirical way for the missing degrees of freedom implied by a single conformer method (see the discussion by Schutz and Warshel [71]).

#### Monte Carlo simulation of the ionization state

The protonation state of the ionizable sidechains is changed according to a Monte Carlo procedure. Protonation or deprotonation are simulated by adding or subtracting a unit charge to an atom representing the ionizable group. The following atoms have been considered as representative for each ionizable group: CG for Asp, CD for Glu, NZ for Lys, CE for Arg, SG for Cys, OH for Tyr, N for the N-terminal ammine and C for the C-terminal carboxyl. For histidine protonation may occur alternatively at the atom NE2 and ND1.

Monte Carlo simulations are performed at 0.5 pH intervals between -2.0 and 18.0 and at each pH value average ionization values and average components of ionization free energy are computed. At each pH value the number of equilibration steps is 100 times the number of ionizable sites and the number of Monte Carlo steps is 1000 times the number of ionizable sites.

Based on Monte Carlo simulation the output of the program includes: i) the titration curve for each ionizable site; ii) the list of pKa values for each ionizable site; iii) the charge state of the protein; iv) the pH-dependent component of the free energy of folding. Compared to the scheme of Antosiewicz et al. we are using the solvent accessible surface (as detailed in the subsection "Solvent accessible surface") instead of the molecular surface because results are more stable.

Heuristic corrections (detailed hereafter) to the scheme of Antosewicz et al [60] have been done mainly to remove large contributions which are typically overestimated due to neglection of molecular flexibility. For instance for surface residues large unfavorable interactions may be accomodated by conformational relaxation. Also large charge-charge interactions are likely to lead to partial unfolding that relieves the strong unfavorable energy. These considerations are consistent with the idea of using different dielectric constants for buried and solvent exposed residues, as suggested many years ago by Demchuck and Wade [72].

#### Scaling self energies

Here we consider as solvent exposed all those sites that have generalized Born radius smaller than 4.0 Å and buried those that have generalized Born larger than 7.0 Å. Self-energy interactions (second right-hand term in Equation 32) are multiplied by a factor 2.0 for buried sites and by 0.25 for exposed residues. For all intermediate situations the multiplying factor is a linear combination of the two extreme values.

#### Scaling background interactions

For exposed residues unfavorable background interactions (third right-hand term in Equation 32) are weighted by the empirical function $\exp\left(-\frac{E}{2RT}\right)$. The factor 2 in the Boltzmann-like function is purely empirical to downweigh a contribution that is likely to be relaxed by protein flexibility.

For each ionizable site the pKa is obtained as the midpoint of the titration curve. The value is further corrected by separating the shift in pKa due to the desolvation self-energy $\left(dpK_{a}^{self}\right)$, background interactions $\left(dpK_{a}^{bg}\right)$ and site-charge - site-charge interactions $\left(dpK_{a}^{ii}\right)$.

#### Final scaling of contributions

The site-charge - site-charge interactions shift is scaled by the function $dpK_{a}^{ii}=\frac{dpK_{a}^{ii}\times 3.0}{|dpK_{a}^{ii}|+3.0}$ which sets 3 pKa units as the maximum contribution arising from interactions between titratable sites, consistent with the idea that large unfavorable interactions lead to partial or global unfolding. Similarly, background interaction shifts are scaled by the function $dpK_{a}^{bg}=\frac{dpK_{a}^{bg}\times 5.0}{|dpK_{a}^{bg}|+5.0}$, which sets 5 pKa units as the maximum contribution due to background interactions, in order to avoid few very large shifts.

### Test sets and conditions

The 55 proteins selected by Tjong and Zhou [31] for testing the GBR6 model have been used here. Atoms of the PDB structures have been assigned charges and radii taken from the CHARMM forcefield, using the program PDB2PQR [61,73]. The PQR file format is described at [52]. The radius of hydrogen atoms was reset to 1.0 Å in order to avoid numerical inaccuracies in the analysis linked to the small radius of polar hydrogens in the CHARMM forcefield. The surface of the molecule was defined alternatively as the surface accessible to the center of a solvent probe sphere with radius 1.5 Å or as the surface accessible by the surface of a solvent probe sphere with radius 1.5 Å, except where noted. The first type of surface, here referred to as solvent accessible surface, is computed by default by the program while the second type of surface, here referred to as molecular surface, was computed using the program MSMS [42] and given as input to the program in the form of a list of vertices and normal vectors.

For the computation of self energies 1000 sites were randomly chosen in the 55 proteins providing an unbiased sample of different environments.

The inner dielectric constant was assumed to be 1.0, the outer dielectric constant was assumed to be 78.54, the temperature is 298.15 K, ionic strength 0.150 M, except where noted. For the computation of pKa of ionizable sites in proteins we used the database developed by Gunner and coworkers [43] available at [74]. The test set includes pKa for structures with PDB id: 1a2p, 1a6k, 1beg, 1bf4, 1bhc, 1bus, 1bvi, 1bvv, 1cdc, 1coa, 1cvo, 1de3, 1dg9, 1dwr, 1egf, 1gb1, 1goa, 1h4g, 1ig5, 1igc, 1kf3, 1lni and 1lse.

### Exact generalized Born radii for a conducting sphere

We analyze the simple planar and spherical systems which are treatable analytically with closed formulae in the hypothesis of grounded conducting surface. We consider a hollow conducting grounded sphere of radius *R *filled with a medium with dielectric constant *ε_in_*. The reaction field for a point charge in the sphere may be easily obtained by the image charge method. The reaction field due to a source charge displaced by *a *with respect to the center of the sphere is computed as the field due to an image charge $q^{\prime }=-q\frac{R}{a}$ at a distance from the center $\frac{R^{2}}{a}$ along the direction from the center to the source charge:

(33) $$U_{i}^{react,sphere}=-\frac{q_{i}}{4\pi \varepsilon_{0}\varepsilon_{in}}\frac{R}{R^{2}-a^{2}}$$

which corresponds to a Born radius:

(34) $$\alpha_{i}=\frac{R^{2}-a^{2}}{R}$$

In the limit of *R *→ ∞ and *a *→ *R *the above expression reduces to:

(35) $$U_{i}^{react,plane}=-\frac{q_{i}}{4\pi \varepsilon_{0}\varepsilon_{in}}\frac{1}{2d}$$

where *d *is distance of the charge from the delimiting plane. The polarization charge contribution to the potential due to a source charge *j *at a point *i *inside the sphere is given by:

(36) $$U_{ij}=-\frac{q_{j}}{4\pi \varepsilon_{0}\varepsilon_{in}\sqrt{r_{ij}^{2}+\alpha_{i}\alpha_{j}}}$$

where *α_i _*is the generalized Born radius at point *i*.

As noted by Grycuk [30] for a charge placed at a distance *a *from the center of a sphere with radius *R*, the Born radius computed according to equation (5) results in:

(37) $$\frac{\hat{1}}{\alpha_{i}}=\frac{1}{2}\left(\frac{R}{R^{2}-a^{2}}+\frac{1}{2a}\log\frac{R+a}{R-a}\right)$$

This provides the exact Born radius in the limit *a *→ 0, i.e. when the charge is placed at the center of the sphere, but underestimates Born radius in the limit *R *→ ∞ and *a *→ *R *(i. e. the plane) by a factor 2. It would be desirable to find a surface integral that recovers the correct expression at least for charges embedded in a sphere. The same surface integral should have a form similar to that of equation (5) in order to ensure that no problems arise from complex shape surfaces, e. g. like those of biomolecules. A convenient correction may be sought considering other integrals like in equation (5) with increasing powers of the distance $\left||{\vec{r}}_{i}-{\vec{r}}_{S}|\right|$. In particular we may define the following integrals:

(38) $$I_{3}=\int_{S}\frac{1}{4\pi }\frac{\left({\vec{r}}_{S}-{\vec{r}}_{i}\right)\cdot \vec{n}\left({\vec{r}}_{S}\right)}{||{\vec{r}}_{S}-{\vec{r}}_{i}|{|}^{4}}dS$$

(39) $$I_{4}=\int_{S}\frac{1}{4\pi }\frac{\left({\vec{r}}_{S}-{\vec{r}}_{i}\right)\cdot \vec{n}\left({\vec{r}}_{S}\right)}{||{\vec{r}}_{S}-{\vec{r}}_{i}|{|}^{5}}dS$$

(40) $$I_{n}=\int_{S}\frac{1}{4\pi }\frac{\left({\vec{r}}_{S}-{\vec{r}}_{i}\right)\cdot \vec{n}\left({\vec{r}}_{S}\right)}{||{\vec{r}}_{S}-{\vec{r}}_{i}|{|}^{n+1}}dS$$

For the sphere these integrals have closed forms that can be obtained using the formal integrator of Mathematica [75]. If the position of the source charge is displaced from the center of the sphere by a quantity *a *and *R *is the radius of the sphere the integrals have the following values:

(41) $$I_{3}=\frac{1}{2}\left(\frac{R}{R^{2}-a^{2}}+\frac{1}{2a}\log\frac{R+a}{R-a}\right)$$

(42) $$I_{4}=\frac{4a^{2}}{6{\left(R^{2}-a^{2}\right)}^{2}}+\frac{1}{R^{2}-a^{2}}$$

(43) $$I_{5}=\frac{R^{3}}{{\left(R^{2}-a^{2}\right)}^{3}}$$

(44) $$I_{6}=\frac{24a^{4}+40a^{2}\left(R^{2}-a^{2}\right)+15{\left(R^{2}-a^{2}\right)}^{2}}{15{\left(R^{2}-a^{2}\right)}^{4}}$$

(45) $$I_{7}=\frac{16Ra^{4}+22a^{2}\left(R^{2}-a^{2}\right)+6R{\left(R^{2}-a^{2}\right)}^{2}}{6{\left(R^{2}-a^{2}\right)}^{5}}$$

The integral *I*_5 _is interesting because, as pointed out by Grycuk [30] it can be easily inverted to get the correct Born radius for both the plane and the sphere:

(46) $$\alpha_{i}={\left(\frac{1}{I_{5}}\right)}^{\frac{1}{3}}$$

The work of Mongan et al. [33] reports a compact form for the Born radii corresponding to the integrals above expressed in term of GBR6 multiplied by a correction factor.

## List of abbreviations used

PB: Poisson-Boltzmann; GB: Generalized Born; COSMO: Conducting Surface Model; RMSD: Root mean square deviation.

## Competing interests

The authors declare that they have no competing interests.

## Authors' contributions

FF, AC, PV, GE conceived the project and tests reported in the manuscript. VY did some of the tests reported. VY and AJ designed a web interface for internal tests and for further development.

## Supplementary Material

Additional file 1 **Executable and examples**. Zipped folder with the executable compiled for Linux x86_64 and 80386 machines and including example input and output files. Click here for file

## References

[B1] Fogolari F Brigo A Molinari H The Poisson-Boltzmann equation for biomolecular electrostatics: a tool for structural biology J Mol Recogn 2002 15 377 392 10.1002/jmr.577 12501158

[B2] Neves-Petersen MT Petersen SB Protein electrostatics: a review of the equations and methods used to model electrostatic equations in biomolecules-applications in biotechnology Biotechnol Annu Rev 2003 9 315 395 14650935 10.1016/s1387-2656(03)09010-0

[B3] Baker NA Improving implicit solvent simulations: a Poisson-centric view Curr Opin Struct Biol 2005 15 137 143 10.1016/j.sbi.2005.02.001 15837170

[B4] Lu BZ Zhou YC Holst MJ McCammon JA Recent progress in numerical methods for the Poisson-Boltzmann equation in biophysical applications Commun Comput Phys 2008 3 973 1009

[B5] Wang J Tan C Tan YH Lu Q Luo R Poisson-Boltzmann solvents in molecular dynamics simulations Commun Comput Phys 2008 3 1010 1031

[B6] Kirkwood JG Theory of Solutions of Molecules Containing Widely Separated Charges with Special Application to Zwitterions J Chem Phys 1934 2 351 361 10.1063/1.1749489

[B7] Fenley AT Gordon JC Onufriev A An analytical approach to computing biomolecular electrostatic potential. II. Derivation and analysis J Chem Phys 2008 129 075101 10.1063/1.2956497 19044802 PMC2671191

[B8] Gordon JC Fenley AT Onufriev A An analytical approach to computing biomolecular electrostatic potential. II. Validation and applications J Chem Phys 2008 129 075102 10.1063/1.2956499 19044803 PMC2671192

[B9] Sigalov G Fenley A Onufriev A Analytical electrostatics for biomolecules: Beyond the generalized Born approximation J Chem Phys 2006 124 124902 10.1063/1.2177251 16599720

[B10] Sigalov G Scheffel P Onufriev A Incorporating variable dielectric environments into the generalized Born model J Chem Phys 2005 122 094511 10.1063/1.1857811 15836154

[B11] Bashford D Case DA Generalized born models of macromolecular solvation effects Annu Rev Phys Chem 2000 51 129 152 10.1146/annurev.physchem.51.1.129 11031278

[B12] Feig M Brooks CL Recent advances in the development and application of implicit solvent models in biomolecule simulations Curr Opin Struct Biol 2004 14 217 224 10.1016/j.sbi.2004.03.009 15093837

[B13] Koehl P Electrostatics calculations: latest methodological advances Curr Opin Struct Biol 2006 16 142 151 10.1016/j.sbi.2006.03.001 16540310

[B14] Chen J Brooks CL Khandogin J Recent advances in implicit solvent-based methods for biomolecular simulations Curr Opin Struct Biol 2008 18 140 148 10.1016/j.sbi.2008.01.003 18304802 PMC2386893

[B15] Still WC Tempczyk A Hawley RC Hendrickson T Semianalytical treatment of solvation for molecular mechanics and dynamics J Am Chem Soc 1990 112 6127 6129 10.1021/ja00172a038

[B16] Lee MS Feig M Salsbury FR Brooks CL New analytic approximation to the standard molecular volume and its application to generalzied Born calculations J Comp Chem 2003 24 1348 1356 10.1002/jcc.10272 12827676

[B17] Onufriev A Case DA Bashford D Effective Born radii in the generalized Born approximation: the importance of being perfect J Comput Chem 2002 23 1297 1304 10.1002/jcc.10126 12214312

[B18] Lee MS Salsbury FR A OM New analytic approximation to the standard molecular volume and its application to generalzied Born calculations J Comp Chem 2004 25 1967 1978 10.1002/jcc.20119 12827676

[B19] Miertus S Scrocco E Tomasi J Electrostatic interaction of a solute with a continuum. A direct utilizaion of AB initio molecular potentials for the prevision of solvent effects Chem Phys 1981 55 117 129 10.1016/0301-0104(81)85090-2

[B20] Zauhar RJ Morgan RS A new method for computing the macromolecular electric potential J Mol Biol 1985 186 815 820 10.1016/0022-2836(85)90399-7 4093987

[B21] Rashin AA Namboodiri K A simple method for the calculation of hydration enthalpies of polar molecules with arbitrary shapes J Phys Chem 1987 91 23 6003 6012 10.1021/j100307a038

[B22] Yoon BJ Lenhoff AM A boundary element method for molecular electrostatics with electrolyte effects J Comp Chem 1990 11 1080 1086 10.1002/jcc.540110911

[B23] Lu B Cheng X Huang J McCammon JA An Adaptive Fast Multipole Boundary Element Method for Poisson-Boltzmann Electrostatics J Chem Theory Comp 2009 5 1692 1699 10.1021/ct900083k PMC2693949 19517026

[B24] Bardhan JP Interpreting the Coulomb-field approximation for generalized-Born electrostatics using boundary-integral equation theory J Chem Phys 2008 129 144105 10.1063/1.2987409 19045132

[B25] Bardhan JP Numerical solution of boundary-integral equations for molecular electrostatics J Chem Phys 2009 130 094102 10.1063/1.3080769 19275391

[B26] Klamt A Schüürmann G COSMO: a new approach to dielectric screening in solvents with explicit expressions for the screening energy and its gradient J Chem Soc Perkin Trans 1993 2 799 805

[B27] Klamt A Eckert F Arlt W COSMO-RS: an alternative to simulation for calculating thermodynamic properties of liquid mixtures Ann Rev Chem Biomol Eng 2010 1 101 120 10.1146/annurev-chembioeng-073009-100903 22432575

[B28] Jackson JD Classical Electrodynamics Third Edition 1998 New York, NY, USA: Wiley and sons

[B29] Ghosh A Rapp CS Friesner RA Generalized Born Model Based on a Surface Integral Formulation J Phys Chem B 1998 102 10983 10990 10.1021/jp982533o

[B30] Grycuk T Deficiency of the Coulomb-field approximation in the generalized Born model: An improved formula for Born radii evaluation J Chem Phys 2003 119 4817 4826 10.1063/1.1595641

[B31] Tjong H Zhou HX GBr^6^: a parametrization free, accurate, analytical generalized Born method J Phys Chem 2007 111 3055 3061 10.1021/jp066284c 17309289

[B32] Tjong H Zhou HX GBr^6^NL: a generalized Born method for accurately reproducing solvation energy of the nonlinear Poisson-Boltzmann equation J Chem Phys 2007 126 195102 10.1063/1.2735322 17523838

[B33] Mongan J Svrcek-Seiler WA Onufriev A Analysis of integral expressions for effective Born radii J Chem Phys 2007 127 185101 10.1063/1.2783847 18020664

[B34] Romanov AN Jabin SN Martynov YB Sulimov AV Grigoriev FV Sulimov VB Surface generalized Born method: a simple, fast and precise implicit solvent model beyond the Coulomb approximation J Phys Chem 2004 108 9323 9327 10.1021/jp046721s

[B35] Hawkins GD Cramer CJ Truhlar DG Parametrized models of aqueous free energies of solvation based on pairwise descreening of solute atomic charges from a dielectric medium J Phys Chem 1996 100 19824 19839 10.1021/jp961710n

[B36] Onufriev A Bashford D Case DA Modification of the generalised Born model suitable for macromolecules J Phys Chem 2000 104 3712 3720

[B37] Onufriev A Bashford D Case DA Exploring protein native states and large-scale conformational changes with a modified generalized Born model Proteins: Struct Func Gen 2004 55 383 394 10.1002/prot.20033 15048829

[B38] Dominy BN Brooks CL Development of a Generalized Born Model Parametrization for Proteins and Nucleic Acids J Phys Chem B 1999 103 3765 3773 10.1021/jp984440c

[B39] Im W Lee MS Brooks CL Generalized born model with a simple smoothing function J Comp Chem 2003 24 1691 1702 10.1002/jcc.10321 12964188

[B40] Yu Z Jacobson MP Friesner RA What role do surfaces play in GB models? A new-generation of surface-generalized born model based on a novel gaussian surface for biomolecules J Comp Chem 2006 27 72 89 10.1002/jcc.20307 PMC2743414 16261581

[B41] Gilson MK Davis ME Luty BA McCammon JA Computation of electrostatic forces on solvated molecules using the Poisson-Boltzmann equation J Phys Chem 1993 97 3591 3600 10.1021/j100116a025 15974723

[B42] Sanner M Spehner JC Olson A Reduced surface: an efficient way to compute molecular surfaces Biopolymers 1996 38 305 320 10.1002/(SICI)1097-0282(199603)38:3<305::AID-BIP4>3.0.CO;2-Y 8906967

[B43] Song Y Mao J Gunner MR MCCE2: Improving protein pKa calculations with extensive side chain rotamer sampling J Comp Chem 2009 30 2231 2247 10.1002/jcc.21222 PMC2735604 19274707

[B44] Li H Robertson AD Jensen JH Very fast empirical prediction and rationalization of protein pKa values Proteins 2005 61 704 721 10.1002/prot.20660 16231289

[B45] Bas DC Rogers DM Jensen JH Very fast prediction and rationalization of pKa values for protein-ligand complexes Proteins 2008 73 765 783 10.1002/prot.22102 18498103

[B46] Olsson MHM Søndergaard CR Rostkowski M Jensen JH PROPKA3: consistent treatment of internal and surface residues in empirical pKa predictions J Chem Theory Comput 2011 7 525 537 10.1021/ct100578z 26596171

[B47] Søndergaard CR Olsson MHM Rostkowski M Jensen JH Improved Treatment of Ligands and Coupling Effects in Empirical Calculation and Rationalization of pKa Values J Chem Theory Comput 2011 7 2284 2295 10.1021/ct200133y 26606496

[B48] Stanton CL Houk KN benchmarking pKa prediction methods for residues in proteins J Chem Theory Comput 2008 4 951 966 10.1021/ct8000014 26621236

[B49] Huang RB Du QS Wang CH Liao SM Chou KC A fast and accurate method for predicting pKa of residues in proteins Protein Eng Des Sel 2010 23 35 42 10.1093/protein/gzp067 19926592

[B50] Burger SK Ayers PW A parameterized, continuum electrostatic model for predicting protein pKa values Proteins 2011 79 2044 2052 10.1002/prot.23019 21557315

[B51] Humphrey W Dalke A Schulten K VMD Visual Molecular Dynamics J Mol Graph 1996 14 33 38 10.1016/0263-7855(96)00018-5 8744570

[B52] APBS - File Formats Http://www.poissonboltzmann.org/file-formats/

[B53] Baker NA Sept D Joseph S Holst MJ McCammon JA Electrostatics of nanosystems: application to microtubules and the ribosome Proc Natl Acad Sci USA 2001 98 10037 10041 10.1073/pnas.181342398 11517324 PMC56910

[B54] Madura JD Davis ME Gilson MK Wade R Luty BA McCammon JA Biological applications of electrostatics calculations and Brownian dynamics simulations Rev Comp Chem 1994 5 229 267

[B55] Madura JD Briggs JM Wade R Davis ME Luty BA Ilin A Antosiewicz JA Gilson MK Bagheri B Ridgway Scott L McCammon JA Electrostatics and diffusion of molecules in solution: simulations with the University of Houston Brownian Dynamics program Comput Commun Phys 1995 91 57 95 10.1016/0010-4655(95)00043-F

[B56] Press WH Teukolsky SA Vetterling WT Flannery BP Numerical recipes in C (2nd ed.) the art of scientific computing 1992 Cambridge, UK: Cambridge University Press

[B57] Srinivasan J Trevathan MW Beroza P Case DA Application of a pairwise generalized Born model to proteins and nucleic acids: inclusion of salt effects Theor Chem Acc 1999 101 426 434 10.1007/s002140050460

[B58] Flanders H Differentiation Under the Integral Sign Am Math Month 1973 80 615 627 10.2307/2319163

[B59] Aris R Vectors, tensors, and the basic equation of fluid mechanics 1962 New York, NY, USA: Dover Publications

[B60] Antosiewicz J McCammon JA Gilson MK Prediction of pH-dependent properties of proteins J Mol Biol 1994 238 415 436 10.1006/jmbi.1994.1301 8176733

[B61] Dolinsky TJ Czodrowski P Li H Nielsen JE Jensen JH Klebe G Baker NA PDB2PQR: expanding and upgrading automated preparation of biomolecular structures for molecular simulations Nucleic Acids Res 2007 35 W522 525 10.1093/nar/gkm276 17488841 PMC1933214

[B62] Simonson T Carlsson J Case DA Proton binding to proteins: pK(a) calculations with explicit and implicit solvent models J Am Chem Soc 2004 126 4167 4180 10.1021/ja039788m 15053606

[B63] Mongan J Case DA McCammon JA Constant pH molecular dynamics in generalized Born implicit solvent J Comput Chem 2004 25 2038 2048 10.1002/jcc.20139 15481090

[B64] Gordon JC Myers JB Folta T Shoja V Heath LS Onufriev A H++: a server for estimating pKas and adding missing hydrogens to macromolecules Nucleic Acids Res 2005 33 W368 371 10.1093/nar/gki464 15980491 PMC1160225

[B65] Myers J Grothaus G Narayanan S Onufriev A A simple clustering algorithm can be accurate enough for use in calculations of pKs in macromolecules Proteins 2006 63 928 938 10.1002/prot.20922 16493626

[B66] Mehler EL Guarnieri F A self-consistent, microenvironment modulated screened coulomb potential approximation to calculate pH-dependent electrostatic effects in proteins Biophys J 1999 77 3 22 10.1016/S0006-3495(99)76868-2 10388736 PMC1300308

[B67] Wisz MS Hellinga HW An empirical model for electrostatic interactions in proteins incorporating multiple geometry-dependent dielectric constants Proteins 2003 51 360 377 10.1002/prot.10332 12696048

[B68] Pokala N Handel TM Energy functions for protein design I: efficient and accurate continuum electrostatics and solvation Protein Sci 2004 13 925 936 10.1110/ps.03486104 15010542 PMC2280065

[B69] He Y Xu J Pan XM A statistical approach to the prediction of pK(a) values in proteins Proteins 2007 69 75 82 10.1002/prot.21478 17588227

[B70] Spassov VZ Yan L A fast and accurate computational approach to protein ionization Protein Sci 2008 17 1955 1970 10.1110/ps.036335.108 18714088 PMC2578799

[B71] Schutz CN Warshel A What are the dielectric "constants" of proteins and how to validate electrostatic models? Proteins 2001 44 400 417 10.1002/prot.1106 11484218

[B72] Demchuk E Wade RC Improving the Continuum Dielectric Approach to Calculating pKas of Ionizable Groups in Proteins J Phys Chem 1996 100 43 17373 17387 10.1021/jp960111d

[B73] Dolinsky TJ Nielsen JE McCammon JA Baker NA PDB2PQR: an automated pipeline for the setup of Poisson-Boltzmann electrostatics calculations Nucleic Acids Res 2004 32 W665 667 10.1093/nar/gkh381 15215472 PMC441519

[B74] Protein pKa database Http://pka.engr.ccny.cuny.edu

[B75] Wolfram Mathematica online integrator Http://integrals.wolfram.com

